# Public trust and ‘ethics review’ as a commodity: the case of Genomics England Limited and the UK’s 100,000 genomes project

**DOI:** 10.1007/s11019-017-9810-1

**Published:** 2017-10-30

**Authors:** Gabrielle Natalie Samuel, Bobbie Farsides

**Affiliations:** Brighton and Sussex Medical School, University of Sussex, Falmer, BN1 9PX UK

**Keywords:** Ethics, Ethics committees, Health policy, Governance, Genomics, Genetics

## Abstract

The UK Chief Medical Officer’s 2016 Annual Report, *Generation Genome*, focused on a vision to fully integrate genomics into all aspects of the UK’s National Health Service (NHS). This process of integration, which has now already begun, raises a wide range of social and ethical concerns, many of which were discussed in the final Chapter of the report. This paper explores how the UK’s 100,000 Genomes Project (100 kGP)—the catalyst for *Generation Genome*, and for bringing genomics into the NHS—is negotiating these ethical concerns. The UK’s 100 kGP, promoted and delivered by Genomics England Limited (GEL), is an innovative venture aiming to sequence 100,000 genomes from NHS patients who have a rare disease, cancer, or an infectious disease. GEL has emphasised the importance of ethical governance and decision-making. However, some sociological critique argues that biomedical/technological organisations presenting themselves as ‘ethical’ entities do not necessarily reflect a space within which moral thinking occurs. Rather, the ‘ethical work’ conducted (and displayed) by organisations is more strategic, relating to the politics of the organisation and the need to build public confidence. We set out to explore whether GEL’s ethical framework was reflective of this critique, and what this tells us more broadly about how genomics is being integrated into the NHS in response to the ethical and social concerns raised in *Generation Genome*. We do this by drawing on a series of 20 interviews with individuals associated with or working at GEL.

## Introduction

On 4th July 2017 the UK Chief Medical Officer (CMO), Dame Sally Davies, published her 2016 Annual Report, *Generation Genome*, which focused on her vision to integrate genomics into the UK’s national healthcare system (Davies 2017). The final Chapter of her report explores how this vision, which she coined the ‘genomic dream’, should be implemented in an ethically and socially acceptable manner. It stresses the need for an appropriate ethical governance system that can be trusted by patients; responsible health professionals which uphold this trust; clear, open protection, and privacy of genomic data; and best practice approaches to patient consent (Lucassen et al. 2017). In this paper we explore how some of these ethical issues are beginning to be addressed in practice. Specifically we explore how the UK’s 100,000 Genomes Project (100 kGP)—the catalyst of this ‘genomic dream’, and catalyst for bringing genomics into the UK National Health Service (NHS), is negotiating some of these ethical concerns.

The UK’s 100 kGP, promoted and delivered by Genomics England Limited (GEL), is an innovative venture aiming to sequence 100,000 genomes from NHS patients who have a rare disease, cancer, or an infectious disease (Gov.uk 2012). Launched in 2012 by ex-UK Prime Minister David Cameron, and due to be completed by 2018,^1^ its ambition reflects that of the CMO’s report (and indeed Dame Sally Davies sits on GEL’s board)—to incentivise the transformation of clinical care so that genome sequencing eventually becomes routine diagnostic practice within the NHS. It also has a research-focused goal to provide genome data for scientific discovery and future patient benefit.^2^ The NHS has been given responsibility for recruiting and consenting patients to the project, and collecting patients’ DNA samples for later clinical and research analysis; GEL’s responsibilities lie with procuring the genome sequencing, and established a bio-repository for data storage and clinical/research analysis by a range of commercial, clinical and academic actors.^3^^,^ 4 It is foreseen that by the completion of the project an NHS infrastructure for genomic medicine, both in terms of clinical and research practice, will have been established and ‘generation genome’ will be underway.

Unsurprisingly, 100 kGP raises ethical questions aligned with those highlighted in the CMO’s annual report, including how best to consent patients to the project (in terms of dealing with additional findings,^5^ confidentiality, and patient expectations); how best to ensure data protection and privacy; how to engage/inform the public about the venture; how to ensure equitable access to care; and how best to interact with commercial companies wishing to access genomic data for research; as well as important questions less spoken about in the CMO’s report around the value and usefulness of incorporating genomics into healthcare more broadly.

Given the range of ethical concerns associated with 100 kGP, and the venture’s importance as a key initiator of integrating genomics into the NHS, GEL has made an open commitment to act ethically in terms of its policies and practice. Drawing on the contemporary belief that engaging the public is synonymous with ethical decision-making (Irwin 2006), it has a strong public engagement strategy comprising a resourceful website and a wide range of events to both inform the public and also to listen to their opinions about how the project is un-folding in practice^6^ (Samuel and Farsides 2017a, b). Furthermore, a bioethicist sits as a non-executive member on GEL’s board to emphasise the ethical dimensions of policy decision-making; and within the organisation GEL has a team of two individuals who are responsible for ensuring commitment to ethical policymaking and adherence to ethical regulations in practice.

Within the range of committees established by GEL to help with the formulation of policy and strategies, those with a particular focus on ‘ethics’ include the participant panel (which ensures patients and the public are involved in decision-making), the public engagement committee (responsible for public engagement activities), and the data access committee (controls access to the data and samples collected within the project). Finally, in line with the reasoning that ethics committees associated with large, complex biomedical organisations allow for concurrent, practical ethical questioning of projects, GEL has also established an independent Ethics Advisory Committee (EAC). The committee meets around four times a year to discuss and advise on any pertinent issues emerging from the venture and is compromised of individuals from clinical and research genetics, policy, law, ethics, nursing, and patient/ public representatives. Three EAC sub-committees give specific advice on the areas of the consent process, issues of equity of access to care/research, and issues of legacy and ethical management once 100 kGP finishes. Given all this, and in line with the CMO’s report to ensure the consideration of ethical and social issues when bringing genomics into healthcare, there is no question that GEL’s framework has been established to accommodate, and even emphasise the importance of ethical decision-making and governance when developing the organisation’s policy and practice.^7^

GEL’s commitment to ethics reflects not only those calls made within the CMO’s report to bring genomics into the NHS in an ethically acceptable manner, but also the contemporary landscape of science governance more generally, in which increasing attention is being paid to ethical issues at the policy level. In fact, Bogner and Menz claim that ‘*ethics has become the dominant discourse [of science governance]. In the course of this ‘‘ethical turn’’ national ethics councils [have been] set up throughout Europe and in the United States to advice politics in ethically controversial issues’* (p. 888) and ethics is ‘*now the relevant discourse of reflection in nearly all areas of society*’ (Bogner and Menz 2010, p.892).

The widespread adoption of this ‘ethical turn’ has led to a new level of sociological critique, where it has been argued that biomedical or biotechnological organisations presenting themselves as ‘ethical’ entities do not necessarily reflect a space within which moral thinking occurs—especially where organisations are politically incentivised, as is the case for GEL. Rather, the ‘ethical work’ conducted (and displayed) by organisations is more strategic such that the moral messaging relates to the politics of the organisation (Petersen 2005; Hoeyer 2012), with the ‘ethical presence’ within the company being mostly about building public confidence and legitimising the organisation (Jasanoff 2005).^8^ As Hoeyer notes in relation to biobanking;

Ethics has left the philosophical chamber and become a parameter of competition among researchers and commercial stakeholders. When a Swedish company, UmanGenomics, sought for venture capital, it benefited from having been promoted in Nature and Science as resting on a more robust ‘ethics model’ than the Icelandic company, deCODE. The point is that the change in scale necessitates new forms of maneuvering in relation to potential public opposition and, indeed, proactive ‘ethics work’ has become a prominent feature of large-scale biobanking.…as ethics turns into a…mode of regulation, the word as such gradually changes its meaning (Hoeyer 2012, p.212).

In fact, say Hoyer and Tutton, the term ‘ethics’ has gained a specific institutionalised purpose in ‘*demonstrating that ethical problems are attended to*’ (Hoeyer and Tutton 2005, p. 386).

Moreover, critics have complained that the ‘ethical turn’ can constrict, ‘close’ or thin ethical discourse to a consideration of only formalised or principled notions of concern (such as, consent, autonomy, privacy); to only notions of concern that can be seen to be reasonably negotiated (Petersen 2005; Strassnig 2008; Hilgartner et al. 2016); or to singular discourses which de-emphasise an ‘ethos of controversies’ (Poort et al. 2013). This, say scholars, leaves little room for integration of more social, personal or cultural aspects of ethics, or moral thinking more broadly.

Applying this critique to GEL is concerning. It would mean that whilst the CMO’s report has called for not only patient/public trust to be *built*, but also *upheld*, the establishment of GEL’s ethical framework may only be responding to the former. This is because whilst it would give the impression of strong ethical governance, this would only serve a strategic purpose rather than applying the moral thinking which patients/public are expecting, as well as trusting GEL to ensue. This could then have implications more broadly, since if respect for patients/public is not upheld at this stage, trust in the *Generation Genome* venture, and in the value of bringing genomics into the NHS as a research-clinical framework moving forward, could be eroded.

We therefore wanted to explore GEL’s ethical framework, especially the discussions conducted within the EAC, to determine if this was the case, and whether the ethical discourse within GEL could be described as ‘thin’ or ‘closed’. We conducted 20 interviews with those working at or associated with GEL—including a large proportion of individuals who sit on the EAC—to explore the nature and role of their ethical discussions within GEL.

## Methods

### Identifying participants

In order to contact those working at or associated with GEL, we sought permission from Board Member and EAC Chair, Professor Mike Parker, After viewing and sharing the project’s proposed rationale and methodology with GEL, Professor Mike Parker granted permission for the project to proceed. First author, GS, spoke with Head of Ethics, Laura Riley, about who it would be best to interview via a convenience sample to ensure a full range of opinions, stakeholders and institutions were garnered about 100 kGP and GEL.

### Recruitment

Potential respondents were recruited in summer 2016, at the approximate halfway mark through the 100 kGP. Invitations requesting participation, including participant information sheets, were emailed to individuals associated with, or those who worked for GEL, including: GEL staff members (plus those involved in public engagement and the project’s evaluation); GEL board members; EAC members; and representatives from the Department of Health, Public Health England, NHS England, and Genomics Medicine Centres (GMCs).^9^ Two follow up emails were sent to non-responding individuals.

20 semi-structured interviews were conducted either by telephone or face-to-face (at a location chosen by the participant). Interviews lasted between 30 and 105 min and were recorded. The interview schedule was broad, asking participants about their background and role with 100 kGP; their views on the project; its benefits (present and potential) and drawbacks; and on the project’s ethics and public engagement strategy. Interviews were transcribed either by GS or by external transcribers. All above categories of individuals were represented in the interviews (exact numbers not provided for confidentiality).

Neither Mike Parker, Laura Riley, nor GEL had any knowledge of who responded to the recruitment emails (and hence who participated in the project). They also had no input into the project design, interview questions which were asked, or the analysis of findings.

### Analysis

Analysis (or coding) (Corbin and Strauss 2008) of interview data was approached using inductive reasoning based on two inter-linked rounds: overview analysis and detailed analysis. Overview analysis consisted of memo-making and broad coding. Extensive memo-making was employed by GS directly after each interview. Broad coding involved scanning the interview transcripts for relevant ideas and themes. Emerging themes identified from memo-taking/broad coding were discussed in detail with second author, BF and it was these discussions which informed early analysis of the data. Once themes emerged from these discussions, detailed analysis of the full transcripts occurred line-by-line by first author, GS, using NVivo software.

## Findings

### GEL’s ethical framework and ‘commodifying’ ethics

GEL’s rationale for implementing an ethical framework was perceived to be related to the extrinsic need to garner public support for the project (Tutton et al. 2004; Williams and Schroeder 2004; Samuel and Farsides 2017a) and nearly all interviewees spoke about the ethical framework constructed by the organisation as value-adding in terms of gaining public trust for the venture: ‘I think it has been clear before GEL was established in the setup phase that it was going to be important for the success of the project for there to be a clear ethical framework that carried public confidence with it’ (interviewee 10). Indeed, the need to be seen to be acting ethically was important to avoid the project’s failure: ‘they’re very important [being seen to be dealing with ethical issues]…if we loose the trust of the public then the project will not only not be a success but is likely to fail’ (interviewee 13). In particular, the visibility of the EAC was perceived as vital to giving patients a reassurance that ethical issues were being handled appropriately (Irwin 2006): *‘*it’s easier when you work with patients saying absolutely there’s an ethics board associated with the project, discussing the issues that patients are bringing up. So I think it’s added validity and credibility*’* (interviewee 11). Being seen to be ethical was in fact a tangible commodity for GEL—perceived to add value to the organisation’s status scientifically, clinically and ethically, and one whose very presence could be exchanged as ‘currency’ for public support and trust.^10^

In positive terms ‘ethics’ can be seen as a valuable commodity within a context where the culture emphasises the importance of accountability, openness and good ethical conduct. However, a more cynical interpretation could present ethics and GEL’s establishment of an ethical framework—and particularly its ‘visible’ EAC—as merely ‘window dressing’ the company with a ‘public-facing’ ethics—the purpose being to ‘sell’ an image that appears to prioritise consideration of ethical issues rather than necessarily addressing them. A more nuanced position might hold that this commodification of ethics is just one-half of a dual-functioning ethical framework—a framework which needs to, on the one hand, be *seen* to be ethical in terms of form and function, and on the other, actually *be* ethical in terms of results. Within this latter perspective, the ethical framework—and especially the EAC—whilst convened to promote GEL as an ethical organisation, creates a secure space in which a group of individuals can think carefully about ethical issues and pave the way for productive ethical discussion (Moore 2010). To provide insight into which model resonates, below we explore GEL interviewees’ views about ethical issues discussed within GEL and the EAC.

### The dual function of ethics: being a ‘commodity’ and ‘being ethical’

Unsurprisingly, all interviewees expressed an intrinsic desire for GEL to make ethical decisions in practice, highlighting the importance placed on the organisation to ‘act ethically’ alongside the need to appear ethical: *‘it’s important that we’re not just ethical but that we’re seen to be ethical as well to provide public confidence and that’s incredibly important’* (interviewee 2). For interviewee 2, and others, it would be: *‘very disappointing if that [being seen to be ethical] was the only purpose that the ethical focus had. I think that that is a necessity of what we need for public engagement but the real important stuff is the ethical questions that we keep on facing as we look at how we address this’*.

In terms of the types of ethical considerations discussed within the EAC, the first point to note is that, as has been documented in terms of other biobank-style initiatives (Wallace 2005), there was no perceived need to question the underlying value or usefulness of the genomics programme within the NHS (a point we return to in the discussion). Rather, interviewees reported discussions were focused on the ethical issues associated with rolling out the venture into practice, and concerns about a range of issues already extensively discussed in the literature, such as how to consent patients; how to ensure that data is secure and confidential; how to interact with commercial companies; how to provide equitable access; and how to handle the clinical issue of additional findings. These discussions were perceived to be where real ethical debates and decision-making occurred, and were seen to be disconnected from the more strategic ethical work used to ‘window dress’ the organisation: *‘I do recognise that there’s probably a disconnect between what actually is presented as the ethics issues and what actually could be recognised to be the ethics issues’* (interviewee 8). We provide two examples of this disconnect to highlight the different types of ethical work which were being conducted at GEL in terms of issues of privacy, commercial interaction and consent.

First, GEL interviewees did not always see those issues which they gave high public prominence to on their websites and during public discussions^11^ i.e., those related to privacy, commercial interaction and data security as the only pertinent issues for them to discuss and reflect on.^12^ Rather, they perceived that whilst some evidence suggests the public attribute more importance to protecting privacy than maximising new healthcare benefits in terms of genetic biobanks (Critchley et al. 2016), this was not the case for patients, whose values were more shaped by the desire to get well/gain a diagnosis. As interviewee 16 noted, it was important to ensure that whist public ethics revolved around broader discussions of privacy, when thinking about GEL’s decision-making the need for a consideration of patients’ social, political and cultural contexts is important, and policies ‘*must be within the reasonable bounds of ethics—this must be determined by the patients who are affected by these diseases and not by entirely well intended but unaffected people who aren’t going through the experience they are’*. In light of this, GEL created a series of online interviews with families who have taken part in 100 kGP^13^ as a way to highlight patient views and beliefs about the project, and to alert GEL to any concerns families may have about their experiences, or about the way the project is unfolding. One such interviewee stated that;

Some people worry that their data will be exposed…or might be used by commercial companies, as far as I’m concerned commercial companies doesn’t [sic] bother me as long as they make benefit to humanity [sic] to people like…my child^14^^,^ 15

Such patient perspectives—where health benefits are prioritised over a consideration of (for example) concerns about interacting with commercial companies—need to be considered alongside other viewpoints, such as those of the public, who, whilst in good health, may be more concerned about privacy and commercial interaction than immediate health benefits.

A second example of the disconnect between public and inward facing ethics emerged from interviewees’ discussions about consent: GEL’s presentation of ethics on its public-facing website, particularly in terms of the slick downloadable consent form and the impression that is created of ethical work having been done, stood in contrast to interviewees’ discussions about the messiness, uncertainty and far from finished issues facing patients when trying to complete the consent form in practice:

That consent form all looks very slick, but it takes a nurse or a clinician over an hour to go through that consent form with the participant. If you think that they have just been diagnosed with cancer for example, are you in that space of mind where you want to be asked all of those questions and go through a very lengthy consent form? I can almost imagine being sat there with a couple of kids around me who are crying and upset and want to go home, but you’re trying to fill in a consent form.

Indeed, EAC interviewees in particular spoke at great length about the range of concerns still troubling them in terms of developing a best practice model for consent, which rather than completed, was considered as an ongoing iterative process between themselves, patients and the research ethics committee who had provided ethical approval for the genomes project. The iterative approach is now visible in the participant information sheets and consent forms, which have recently been re-drafted in response to patient feedback. This includes changes to their layout, a simplification of language, and the addition of illustrative diagrams.

These examples suggest that, in comparison to public facing ethics, the ethical work occurring within the EAC (and within GEL more broadly) recognises the messiness of implementing 100 kGP into practice, and is attuned to the needs and experiences of patients. Though there was definitely some cause for concern: the need to appear ethical was perceived by some interviewees to overshadow the inward facing ethics. As interviewee 18 stressed: ‘*I think our academic, our research bodies, our large communities, our local authorities are far more concerned with telling people about governance, structures, who sits on what committee and so on as opposed to what it is we’re going to do by when’*. Moreover, the ethical work being conducted within GEL, and specifically the EAC, resonates with similar considerations previously discussed in conversations within biobank organisations and their ethical advisory committees. Here several sociological scholars questioned their value, arguing that whilst they do consider the consent process and a range of patient views, they frame ethics too narrowly using only formalised notions of consent, autonomy, and privacy (Petersen 2005) and fail to contextualise ethics within everyday practice.

There was some evidence of this at GEL, for example in their prioritising the importance of the consent process as a central formalised ‘ethical’ aim of the organisation (“*to create an ethical and transparent programme based on consent”*). Moreover, it is well known that decision-making during the consent process is often less related to the information provided (which may not even be read/remembered by patients/participants), and more about patient/participants personal, cultural and social-economic circumstances; their pre-formed expectations about the research; and importantly, the trusting and communicative relationship built between themselves and researcher/clinician (Hoeyer and Lynoe 2006; Hallowell et al. 2010; Grady 2015; Samuel et al. 2017). Whilst GEL aimed to be open and transparent about the project and its expectations, rather than reflecting on the decision-making process in its social context, and the role of the consent process more broadly in terms of decision-making, GEL took the approach of providing extensive information about the project during the consent process as a proxy (albeit one of many) for acting ethically.

### The perceived value of the ethics advisory committee

Interviewees had different perceptions about the value of any of the discussions they had about the ethics associated with 100 kGP within the EAC, and the value of the EAC more broadly in terms of how much their discussions influenced GEL’s policies and practices. Some interviewees viewed the EAC’s role as valuable in raising ethical issues since, ‘*a lot of the them [ethical issues] have come up for at least some sort of discussion in the advisory group, so I think there’s a forum in which they can be raised, and are’* (interviewee 1). For others, the types of discussions being had within the EAC missed certain ethical perspectives. Reflecting on another organisation’s ethics committee, interviewee 5 commented that:

The good thing about that committee was they had a good patient rep side…It was a mixture of people who are rare disease patients who had a certain model of it, and those who were from these disability rights background, you had both…And I think sometimes, that’s not what we’re getting in the 100,000 Genomes Project…we have the medical model much more than we have had the disability model.

Moreover, the lack of consensus reached during EAC discussions, and the fact that policy-making was seen to take place elsewhere, meant the committee was not always perceived to serve any useful function:^16^

[My role is to] turn up at meetings three times a year. I mean its very interesting, I basically don’t think we serve a function. I really am not convinced that we serve any useful function. We discuss things, the people around the table are very nice, we say things. And I’m sure they take notice of the odd things that we say because it is a very competent group of knowledgeable individuals. But I think all the decisions are taken elsewhere (interviewee 3).

Interviewees explained how the EAC’s Chair, who was well-respected in the field and had decades of experience (including the social and ethical contexts of genomics), had mapped the ethical issues before the project’s commencement:

[The Chair] has been involved in the ethics underpinning genetics applied to patients for, well, almost…20 years. So this hasn’t come out of the blue. It’s been evolving gradually and thoughtfully and he’s been thinking quite seriously about what all these issues are for a long period of time (interviewee 7).

Because of this, and because the Chair also sat on GEL’s board, interviewees felt that the project would probably manage very well without the EAC: *‘I think the project could run very well without it [the EAC], with a few people on the executive like [the Chair] who has a lot of integrity’* (interviewee 11).

Others disagreed, insisting that the EAC was only ever supposed to be an advisory group [*‘we don’t have any executive function… ours is advisory up the GEL food chain’* (interviewee 6)], and that its function emanated from acting as a sounding board for the Chair, to give confidence to him when bringing ethical dimensions to the policy-making table:

I have occasionally not been able to make a particular meeting…I come along to the next one and it doesn’t feel like I’ve missed anything…On the other hand, it may well be that [the Chair] doesn’t feel that way, and he actually needs the sounding board, and he needs the authority of having taken it to the committee, to then go back to the board of Genomics England and say well, our advice is this (interviewee 13).

## Discussion

We have explored how ethical issues associated with 100 kGP are being negotiated by GEL as it moves towards establishing genomic medicine as part of NHS infrastructure—an infrastructure supported and promoted by the 2016 CMO Report *Generation Genome*. We have shown that the public presentation of GEL’s ethical framework has acted as a commodity to be exchanged for public trust, and to legitimise the organisation to ensure support for its activities, and the move towards a genomic NHS. The public presentation of ethics gives the impression that ethics is ‘attended to’ and resolved—an impression which critics may construe as being more about fulfilling a policy agenda than providing a space for any real philosophical analysis (Hoeyer and Tutton 2005). As Hoeyer and Tutton note, presenting ethics in this way gives a false impression of what ethics really amounts to in practice and allows for the fact that ‘*no matter how ambitious or controversial, biomedical research is then able to progress*’ (Hoeyer and Tutton 2005, p. 386). Moreover, commodifying ethics has also been argued to prioritise formalised ethical issues (e.g. privacy, consent and autonomy) at the expense of more everyday patient concerns (Petersen 2005; Strassnig 2008).

We have also shown that GEL’s ethical framework, and in particular the EAC, provides a genuine space for ethical discussion. This ethical discussion sat in contrast with the public presentation of ethics, illustrated the messiness of ethical-decision making in practice, and demonstrated GEL’s attentiveness to patient and family members experiences and social setting. However, there were some areas of concern. First, relating to the relevance and purpose of the EAC discussions: having discussions was perceived as worthy, but there was a sense that the debates had during EAC meetings may have little relevance to policy and practice. Second, relating to the medicalised model adopted during EAC discussions: as one interviewee noted, the disability model was lacking in the EAC, which, if included, could have emphasised an interesting discussion about the relevance and usefulness of genomics within the NHS. However, the dual role for the EAC Chair as a GEL board member was perceived as beneficial and meant that a link was provided between EAC discussions and policy decision-making practice. And whilst some interviewees were hesitant, the hope was that this link would allow the former to influence the latter. Though we note that more research would be needed to determine specifically how much dialogue has moved from the EAC to the policy level, and stress that this research is imperative since, with patients placing so much trust in the project, GEL has an ethical responsibility to be mindful of what this ‘trust’ means,^17^ and ensure that the venture does not only gain public trust but that it upholds it (and they are not only having inward ethical discussions, but that these discussions are listened to and acted upon by policy makers). As the Chief Medical Officer’s report states: ‘*the ability of the NHS to show that it can be trusted on these [aforementioned ethical] issues will be an important foundation for the reasonableness of the new social contract [between the NHS and patients with respect to genomics] that we [the authors of the Chapter] propose’* (Davies 2017, Chap. 16 p. 11). A similar point is also emphasied by Woolley and colleagues, who note that ‘*in order to merit and garner trust, guardians of citizens’ health data ought to ensure that they respect the values of the people who are expected to trust them with their data’* (Woolley et al. 2016) and Dame Fiona Caldicott (National Data Guardian),^18^ who notes that there should be ‘no surprises’ to patients about how their genomic data has been used.

Indeed, there was an impression that this trust was upheld, and interviewees perceived GEL to be attentive to the ethical and social issues related to 100 kGP. However, there were at least some serious concerns from interviewees that GEL was prioritising the need to be viewed as ethical rather than actually being ethical. A discrepancy also emerged as we moved away from how GEL negotiated fomalised ethical issues to how such formalised issues were being questioned. This was most clearly evidenced with relation to consent. Here, GEL interviewees’ concerns amounted to achieving a process which best-informed patients about 100 kGP in a clear and understandable way, taking into account patient/participant views, along with their social setting. Less frequently discussed during interviews were questions about the usefulness or appropriateness of the consent process. Other ethical questioning seemed to also be missing, with GEL interviewees noting a limited ethical discussion regarding 100 kGP’s moral worth and its scientific validity and applicability—a point also missing in the CMO Report. We explore these missing discussions below.

It has been argued that space should be made for debates about a biotechnology’s scientific validity or applicability (i.e., whether the technology is fit for function) within an organisation’s practice (such as within an ethics advisory committee), otherwise, implications drawn from any ethical debate would rely on incorrect underlying assumptions about the science itself. As Wallace and Barbour argue in terms of biobanking, removing the debate about whether the science can offer anything useful in terms of patient benefit invalidates any proceeding ethical discussion (Barbour 2003; Wallace 2005). Hoeyer and Tutton (Hoeyer and Tutton 2005) explain why organisations may be hesitant to have this debate, since any further discussion would;

Undermine [the ethics advisory committee’s] very mandate; their own ‘space of action’ would dissolve if they were to conclude that the money intended for UK Biobank would be better spent on public health measures of another kind. To confirm their own space of action, such ethics advisory boards must confirm the importance of research, and limit themselves to identifying the ways it should continue (p. 393).

Whilst this is a point well made, we suggest, as we have done elsewhere (Samuel and Farsides 2017a) that it is not the place for GEL to debate the ethical dimensions of their very existence. It has passed the point where it would be productive for GEL to demonstrate their worth and/or defend their existence in terms of abstract argument. Rather the time has come to demonstrate how they will contribute to health in a scientifically, clinically and ethically robust manner. Discussions about whether or not technologies should be implemented into practice (what has been called the ‘ethics of innovation’ (Strassnig 2008), or the ‘bioethics of innovation’ (Lipworth and Axler 2016)) are a vital aspect of ethical discussion—and an aspect in desperate need of attention. But the place to have such discussions is not necessarily within the organisation which has been given the political green light to go ahead—an organisation which now needs to put its efforts into negotiating the ethical issues the project raises, and under a huge time constraint imposed by the time-limited political expectations (Samuel and Farsides 2017b). Rather, what is needed is a discussion about who should have the responsibility for these wider debates more broadly. This might become increasingly difficult in the wake of the CMO 2016 Report because, by driving Generation Genome at such a pace, this Report leaves little room for broader stakeholder and public discussions which may question the appropriateness of genomic technologies within the healthcare system.

We can also apply a similar argument to the notion of consent: a number of practical reasons made it extremely difficult, logistically, for GEL to question the notion of providing anything other than an information-focussed consent process as the primary emphasis of patient decision-making. These included the pace of the project, the need to get participants recruited, and the consequent time restrictions attached to this, leaving limited time for any form of discussion of different ways to approach consent. Furthermore, GEL’s decision to submit 100 kGP for ethical approval meant that research ethics committee members—adhering to traditional notions of needing to provide all relevant information in the participant information sheet and consent form—placed restrictions on the form’s content. Whilst all of these factors made it difficult to question the notion of the consent process more broadly, GEL did have strategies in place to ensure consent was as patient-led as possible, that GEL maintained open communication channels with patients and the public, and provided a strong ethical governance structure for the whole organisation. This meant that the process did not just act as a proxy for good ethical practice, but as one ‘*important component of an ethics ecosystem’*—something suggested as crucial by the CMO Report’s ethical guidance (Davies 2017, Chap. 16 p. 5). Having said this, it still leaves questions remaining about how best to ensure public/patient interests and desires are adequately met as we move at a fast-pace towards a new form of genomic healthcare, and whose responsibility it is to find the time and space to explore these questions about the usefulness and purpose of consent in an era of genomics, and apply recommendations of best-practice.

## Concluding remarks

GEL’s ethical framework, whilst a commodity established to be exchanged for patient and public trust, seems to offer value in terms of ethical decision-making—conforming to organisational notions of ethical practice, as well as considering, to some extent, more everyday issues which may affect patients. Though we note it is important that GEL (and in time, as *Generation Genome* unfolds, the NHS) remains vigilant to ensure that its desire to ‘window dress’ ethical issues to gain public support does not overshadow the need to *be* ethical. We also note that whilst GEL stopped short of questioning the value of 100 kGP, or moving beyond formalised notions and discussions of ethical practice, this is ethically justifiable. Nevertheless, again, we emphasise the importance of having these types of discussions elsewhere, and pose questions about upon whom this responsibility should fall as the UK moves towards *Generation Genome*, and as genomics becomes more integrated within the UK NHS. Without these discussions, debate around genomics within UK healthcare will remain only within organisational settings, the remit of which (we have argued in this paper) justifiably is much narrower and more fomalised. In which case, there is a risk that the discussions about genomics within the NHS will fall into the trap of being too ‘closed’ and lacking in an ‘ethos of controversies’ (Petersen 2005; Strassnig 2008; Poort et al. 2013; Hilgartner et al. 2016).

## Limitations

Our findings are based on interviewees’ perceptions of ethical decision-making within GEL and give a broad picture of GEL’s practices with relation to ethics, providing valuable insight into the workings of ethics within GEL. However, an ethnographic analysis would be required to explore the ethical work conducted within this organisation in more detail, and to understand the discursive processes and micro-politics which shape the ethical debate during group meetings (at policy and EAC level), as well as how such processes, in turn, influence any ‘digestible output’ which then gets used during policymaking (Strassnig 2008). Moreover, further work, needs to be conducted to compare those ethical issues described by GEL interviewees with the views of health care professionals and NHS patients involved with the genomes project.

## Footnotes

1 Originally 2017, but extended to 2018 due to challenges with recruitment.

2 For more of a discussion about the clinical/research hybrid nature of GEL, see Dheensa et al. *The research and clinical practice distinction in genomic medicine and the 100,000 genomes project: time for a new framework?* Forthcoming.

3
https://quarterly.blog.gov.uk/2014/01/30/100000-genomes/.

4 The genomes data is being stored in a bio-repository. At present GEL maintains ownership of the genomes data, and access is on a ‘borrowing’ basis. There is uncertainty about what will happen to this data post-project.

5 Findings not related to the reason for the test, for example, risks for hereditary cancers that are amenable to risk-reducing interventions but which are unrelated to the presenting condition.

6
http://www.genomicsengland.co.uk/about-genomics-england/.

7 This was deemed especially important in light of the public backlash towards previous ventures that involved the sharing of health data, for example care.data (Carter et al. 2015; Sterckx et al. 2016).

8 Interestingly, Levitt and Weldon’s research on genetic databases suggest that having a ‘public ethics’ does not serve its purpose of gaining trust: ‘*it seems that public consultation and the language of openness and transparency may not be sufficient to establish trust in the governance of genetic databases’* (Levitt and Weldon 2005, p. 311).

9 There are 13 NHS GMCs throughout the UK, each of which are centres of genomics excellence. They are responsible for delivering the genomes project in terms of patient recruitment and consent; and DNA collection, extraction, and transport for sequencing.

10 Others have also talked about notions of “currency” in the moral economy of biotechnology (Petersen 2015).

11 We attended several public debates, and the privacy issue was a predominant discussion point.

12
http://www.genomicsengland.co.uk/about-genomics-england/.

13
https://www.genomicsengland.co.uk/taking-part/participant-stories/.

14
https://www.genomicsengland.co.uk/.

15 We have discussed elsewhere that given these videos often display families who are positive about the project or have received a positive result, GEL must be vigilant that they do not act as a conduit for promoting high expectations about the project, which could in turn raise false hope for families about what 100kGP is able to achieve in clinical practice.

16 In contrast, Bogner and Wetz note how ethics is in fact distinguished by a lack of consensus, and this aspect is viewed as its worth—bringing diverse viewpoints to policy deliberation (Bogner and Menz 2010).

17 We have argued elsewhere that respecting trust should be formalised and considered on par with other ethical obligations such as providing information (Samuel et al. 2017).

18
https://www.gov.uk/government/speeches/protecting-confidentiality-and-improving-care-not-a-zero-sum-game?utm_content=buffere0b53&utm_medium=social&utm_source=twitter.com&utm_campaign=buffer.
